# Energy Consumption Structure and Influencing Factors of Farmers in China from the Perspective of Labor Transfer

**DOI:** 10.3390/ijerph20021430

**Published:** 2023-01-12

**Authors:** Jiaojiao Wu, Chen Qing, Wenfeng Zhou, Shili Guo, Dingde Xu

**Affiliations:** 1 Institute of Geographic Sciences and Natural Resources Research, Chinese Academy of Sciences, Beijing 100101, China; 2 University of Chinese Academy of Sciences, Beijing 100101, China; 3 UNEP-International Ecosystem Management Partnership (UNEP-IEMP), Beijing 100101, China; 4 College of Management, Sichuan Agricultural University, Chengdu 611130, China; 5 College of Economics, Southwestern University of Finance and Economics, Chengdu 610074, China; 6 Sichuan Center for Rural Development Research, Chengdu 611130, China

**Keywords:** off-farm employment, energy structure, the countryside, China

## Abstract

Under the background of carbon peak and carbon neutralization, the transformation and upgrading of energy consumption structure is crucial to achieve sustainable environmental development. Based on the questionnaire data of 1080 farmers in Sichuan province in 2021, the IV-Probit model was used to explore the impact of labor from off-farm employment on farmers’ energy consumption structure and its specific mechanism. The results show the following: (1) the overall proportion of off-farm employment is not high, only 23%; in cooking energy, the most farmers use high-quality energy, accounting for up to 94%; (2) in addition to high-quality energy, off-farm employment of labor force is positively and significantly correlated with the remaining six types of energy consumption structure. The results of a heterogeneity analysis show that the proportion of off-farm employment of farmers with a high education level and above has the greatest positive effect on the use of high-quality energy; (3) the results of the mediating effect show that the off-farm employment can affect the energy consumption structure of farmers through the two paths of annual cash income and population structure.

## 1. Introduction

Under the background of carbon peak and carbon neutralization, the transformation and upgrading of energy consumption structure is crucial to achieve a sustainable development of the environment [1,2]. At present, many countries have proposed carbon neutrality targets. For example, the United States and the United Kingdom have proposed to achieve carbon neutrality by mid-century [3,4]; Japan has also pledged to reduce GHG emissions by 46% by 2030 compared to 2013 levels. Most of them adjust their energy structure to achieve carbon neutrality [5,6]. China has also actively promoted the implementation of dual carbon targets in recent years. In the 20th CPC National Congress in 2022, General Secretary Xi Jinping pointed out that we should actively and steadily promote carbon peaking and carbon neutrality, implement carbon peaking actions in a planned and step-by-step manner, and actively participate in global governance to address climate change.

Rural energy is an important part of the whole energy system [7], and cooking energy occupies an important position in rural energy. Studies show that at present, most urban residents use new energy such as electricity and natural gas for lighting, cooking heating or bathing, etc., while traditional biomass energy such as firewood and straw is still the most important living energy for rural residents in most developing countries [8]. For example, about 30% of Chinese people are still unable to cook with safe and efficient energy such as natural gas and liquefied petroleum gas, and 307 million people rely on biomass energy as the main cooking fuel [9]. The consumption of a large amount of traditional biomass energy not only pollutes the rural environment, but also threatens the health of rural residents. For example, Jagger and Shively (2014) found in their study on Uganda that highly polluting energy sources such as firewood and straw would cause severe air pollution, which would lead to birth weight loss and malnutrition, and increase the probability of coughing and breathing difficulties in adults [10]. It even causes diseases such as lung cancer and blindness [11]. The issue of living energy is the key to the quality of life of rural residents. Changing the traditional backward energy consumption mode to a safe, convenient and efficient energy consumption structure is an important prerequisite for the sustainable development of rural areas [12]. Therefore, it is of great significance to study rural energy issues.

In recent years, with the transfer of rural surplus labor to cities, the income, demographic structure, consumption concept and environmental awareness of farmers have undergone significant changes, which further affect the choice and use of living energy by rural residents [13]. Studies have shown that off-farm employment of labor greatly improves the energy consumption structure in rural China and improves the living quality and level of farmers [14]. At the same time, some migrant workers who work in off-farm jobs in cities make money in cities and improve energy facilities at home after returning to the countryside. Therefore, a scientific and reasonable analysis of rural energy use and labor off-farm employment is of great practical significance for China’s sustainable energy development and the transformation of rural energy structure. 

From the existing research, more and more scholars have begun to pay attention to and study China’s rural energy issues. Some scholars have found that family education level, Internet use, off-farm employment and households with members living in urban areas have a positive and significant impact on the choice of clean cooking energy [15]. For example, Zi et al. (2021) found that “gas + electricity” has become a common cooking energy combination mode in rural areas of Henan Province, with 33% of farmers using this combination of energy for cooking [16]. Jiang et al. (2021) found that subjective norms, perceived behavioral control and habits have significant impacts on residents’ behavioral intentions [17]. Ma et al. (2019) found that off-farm income promoted the change in the energy structure of farmers, significantly increasing the use of electricity and natural gas and reducing the use of traditional biomass energy [13]. At the same time, some scholars have found that labor transfer may cause a shortage of agricultural labor, and thus lead to the overuse of fossil fuels in agricultural production [18]. 

In general, existing studies have paid great attention to rural energy issues, but there are still the following shortcomings: First, scholars mostly discuss household income and expenditure of farmers, energy prices and policies, and few scholars pay attention to the consumption structure of rural energy from the perspective of off-farm employment of labor [19]; Secondly, scholars mostly use mid-macro statistical data to study the rural energy consumption structure from the national and regional perspectives [20,21], few scholars make quantitative analysis of micro data and deeply investigate the correlation between it and off-farm employment of labor force. Thirdly, many scholars failed to effectively overcome the endogeneity problem caused by causality between variables, and failed to accurately reveal the quantitative relationship and action mechanism between core variables. Fourth, the current academic circle focuses on energy research from clean energy, commercial energy and other aspects [15,22]; few studies have comprehensively analyzed the correlation between traditional biomass energy and commodity energy, as well as various combinations of energy use and off-farm employment.

Based on this, using the questionnaire data of 1080 farmers in Sichuan province in 2021, the IV-Probit model was used to deeply analyze the correlation between labor off-farm employment and household energy structure, and the intermediary effect model was further used to analyze the mechanism of action between the two. Compared with previous studies, this study has the following four marginal contributions: (1) using the micro data of farmers, it overcomes the inaccuracy of the medium and macro data and improves the credibility of the results; (2) comprehensive analysis of the common rural biomass energy and commodity energy, as well as the relationship between various energy combinations based on the above energy basis and labor off-farm employment; (3) the endogeneity problem of core variables was solved by using instrumental variable method, which further improved the accuracy of result estimation; (4) the mediation effect model is used to empirically analyze the internal mechanism of annual cash income and population structure between off-farm employment and rural energy structure.

## 2. Data and Methods

### 2.1. Data Source

This study mainly uses the questionnaire survey data conducted by the research team in six districts and counties of Sichuan province (Pengzhou, Jiajiang, Luxian, Yuechi, Gao and Nanjiang) from August to October 2021. Sichuan province is a province with a large population in China, with diverse terrain and large differences in income levels among different districts and counties, which has good typicality and representativeness. The survey involved 6 districts and counties, 18 towns and 54 villages in Sichuan province, including two plains, two hills and two mountain counties, respectively. The survey method was a one-on-one, face-to-face interview, and general random sampling and stratified probability random sampling were used to determine the survey samples. The research content mainly includes energy application, farmer’s environmental perception, family livelihood, etc. A total of 1080 valid household questionnaires were obtained.

### 2.2. Methods

#### 2.2.1. Benchmark Model Construction

The objective of this study is to explore the relationship between off-farm employment and household cooking energy use and its mechanism. Since the use of household cooking energy is a dichotomous variable, the Probit model is intended to be used for estimation:

(1) $$Prob\left(Y_{i}=1\right)=\Phi \left(\beta_{0}+\beta_{1}T_{i}+\beta_{2}X_{i}+\varepsilon_{i}\right)$$

In Equation (1), $Y_{i}\;$ is the dependent variable, whose value is 1 means that farmers use this kind of energy, and 0 means that farmers do not use this kind of energy. $T_{i}$ is the core explanatory variable of this study, the off-farm employment of labor force, which is expressed by the ratio of the number of family labor force going out to the total number of families. $X_{i}$ is the control variable, $\beta_{0}$ is the constant term, $\beta_{1}$ and $\beta_{2}$ are the parameters to be estimated of the model and $\varepsilon_{i}$ is the error term.

However, there may be a mutual causal relationship between the off-farm employment of labor force and the use of energy of farmers, thus making the off-farm employment of labor force an endogenous variable, and the above model cannot solve the problem of endogeneity. The IV-Probit model will be used to estimate:

(2) $$Prob\left(Y_{i}=1\right)=\Phi \left(\beta_{0}+\beta_{1}IVT_{i}+\beta_{2}IVX_{i}+\varepsilon_{i}\right)$$

In Equation (2), all variables have the same meaning as (1), and the whole model is realized by Stata 16.0. 

#### 2.2.2. Mediation Effect Model Construction

There are many methods to test the mediating effect, such as stepwise regression coefficient method, Sobel test and Bootstrap test. This study intends to use stepwise regression method to test the mediation effect, and the estimated equation is as follows:

(3) $$Prob\left(Y_{i}=1\right)=\Phi \left(\beta_{0}+\beta_{1}IVT_{i}+\beta_{2}IVX_{i}+\varepsilon_{i}\right)$$

(4) $$M_{i}=\alpha_{0}+\alpha_{1}T_{i}+\alpha_{2}X_{i}+\varepsilon_{i}$$

(5) $$Prob\left(Y_{i}=1\right)=\Phi \left(\gamma_{0}+\gamma_{1}IVT_{i}+\gamma_{2}IVX_{i}+\gamma_{3}IVM_{i}+\varepsilon_{i}\right)$$

In the above formula, $M_{i}$ is the intermediary variable, and other variables have the same meanings as Equation (1). In Equations (2) and (4), the dependent variable is whether farmers use this kind of energy, and the IV-Probit model is still used to estimate the parameters. In Equation (3), the dependent variable is the intermediary variable, namely the annual family income and the family population structure. In this case, the ordinary least square method is adopted.

### 2.3. Selection of Model Variables

The purpose of this study is to explore the impact of labor off-farm employment on farmers’ energy consumption structure and its specific mechanism. Specific measures of various variables are as follows:

#### 2.3.1. Dependent Variable

The dependent variable of this study is the energy consumption structure of farmers. Referring to Ma et al. (2019) and Tian et al. (2020) [12,13], the energy consumption structure of farmers is divided into three types: non-commodity energy (including firewood and straw), low quality energy (including coal and biogas) and high quality energy (including electricity, natural gas and liquefied petroleum gas). If a farmer uses one of the three types of energy, the farmer is considered to have used that energy. For example, if farmers use coal, they are considered to be using low quality energy. Through the continuous combination of the three types of energy, 7 categories of dependent variables can be obtained, respectively: use only non-commodity energy, use only high-quality energy, use only low quality energy, use both non-commodity energy and high quality energy, use both high commodity energy and low quality energy, use both non-commodity energy and low quality energy, use both non-commodity energy and high commodity energy and low quality energy.

#### 2.3.2. Focus Variable

The core independent variable of this study is off-farm employment of labor force, that is, the ratio of labor force employed by households to the total number of households. According to the statistical caliber of the National Bureau of Statistics, off-farm employment refers to the labor force that has worked outside the country for at least 6 months or more in a year, excluding the labor force that has worked outside the country for less than 6 months. 

#### 2.3.3. Instrumental Variable

In fact, there is a mutual causal relationship between the off-farm employment of labor and whether farmers use the above seven types of energy. On the one hand, the off-farm employment of labor forces will change the income structure of families (the increase in wage income leads to the increase in annual cash income of families) and the population structure in rural areas, thus affecting the energy consumption structure of families. On the other hand, household energy consumption structures will also affect labor off-farm employment. For example, labor may use high-quality energy for the health of family members, and the high cost of using high-quality energy encourages labor to migrate to work. In order to solve the possible endogeneity problem of the model, referring to Shao et al. (2021) and Xu et al. (2019), the proportion of off-farm employment of other labor in the same village is taken as the instrumental variable of off-farm employment of labor [20,23]. It is mainly based on the following two considerations: on the one hand, the off-farm employment of farmers is inevitably driven by the off-farm employment of other farmers in the same village, which makes the endogenous variables and instrumental variables highly correlated and meets the correlation requirements; on the other hand, there is no direct correlation between the off-farm employment of other farmers in the same village and the energy consumption structure of the interviewed households. They are independent of each other and theoretically meet the requirement of exclusivity. 

#### 2.3.4. Mediator Variables

Based on the above analysis, off-farm labor employment may influence farmers’ use of cooking energy through household annual income and demographic structure [24,25]. Therefore, this study selected household annual cash income and population structure as the intermediary variables to investigate the intermediate transmission mechanism of labor off-farm employment on household cooking energy structure. Among them, the annual cash income of the family refers to the annual cash income of the surveyed farmers in 2020. The family population structure is measured by the proportion of the family’s dependency and support, that is, the proportion of the number of old people and children in the total family population [26].

#### 2.3.5. Control Variables

This study referred to the literature of Cheng et al. (2018), Hou et al. (2018) and Zhang et al. (2018) [16,19,27] and added some factors that may affect the energy structure of farmers’ cooking as control variables, including individual characteristics, family characteristics and farmers’ cognition [19,26,27]. Personal characteristics include gender, age and years of education; the family characteristics were represented by the distance from home to market, the area of farmland under operation and the floor area of houses. Farmers’ cognition includes farmers’ cognition of their own health status, whether they often pay attention to climate change and other information, and how much they attach importance to the honorary titles such as “Civilized sanitation Demonstration Household” and “clean farmer household” by the government. Household cognition was measured 1–5 on a Likert scale. The description of variables is shown in Table 1.

## 3. Theoretical Analysis and Research Hypotheses

**Figure 1:**
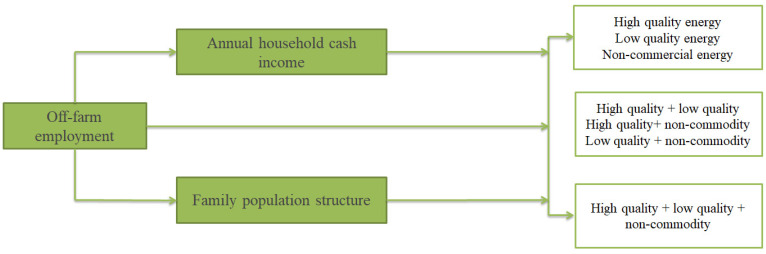
Conceptual framework.

In recent years, China’s economy has grown rapidly. With the improvement of agricultural productivity and the increase in urban enterprises, the rural surplus labor realizes the reallocation of labor resources by migrating to cities. Off-farm employment of labor has played an important role in improving the quality of life, narrowing the gap between urban and rural areas and realizing rural revitalization [13]. Figure 1 shows the conceptual framework of this study. Due to the household registration system and the employment discrimination of migrant workers, a large number of rural labor force can only engage in self-employment in cities or work in non-public units, which determines that they are more inclined to “earn money in the city and go back to the countryside to consume” life mode. The study found that the optimization of cooking energy is an important condition to determine the quality of life, and the transfer of rural labor force changes the consumption pattern of cooking energy in rural areas, thus improving the quality of life of farmers [13,25,28]. Theoretically speaking, off-farm employment will improve farmers’ awareness of environmental protection and consumption, and the improvement of labor value will also encourage them to choose more efficient, convenient and safe cooking energy, and give up the use of traditional biomass energy such as firewood, straw, coal, biogas and other environmentally polluting and inefficient energy. Based on this, research hypothesis H1 is proposed as follows:

**H1:**  *Off-farm employment of labor force has a significant positive correlation with the use of high-quality energy by farmers, and a significant negative correlation with the use of other types of energy*.

Studies have shown that off-farm employment significantly increases the income of migrant workers and improves household energy consumption structures [29]. Off-farm employment increases the household income of farmers, thus reducing the consumption of traditional biomass energy in households and improving the use of high-quality environmental energy such as electricity and natural gas. Based on this, research hypothesis H2 is proposed as follows:

**H2:**  *The off-farm employment of labor forces is mediated by the annual cash income of peasant households, which has a positive and significant effect on the use of high-quality energy by peasant households, and a negative and significant effect on the use of other types of energy.*

After the massive migration of rural labor to cities, the elderly and children stayed at home [24]. First of all, influenced by traditional ideas, the elderly think firewood and straw are easy to obtain, the cost is low, and the food made is more delicious. Secondly, the elderly have a weak ability to learn knowledge and it is difficult to accept new things and the cost of using high-quality energy such as electricity and natural gas is high. Therefore, the elderly are more inclined to use traditional biomass energy or other low-quality energy [30]. Based on this, research hypothesis H3 is proposed as follows:

**H3:**  *The off-farm employment of labor has a significant negative effect on the use of high-quality energy and a significant positive effect on the use of other types of energy, with the dependency/support ratio of households as the intermediary.*

## 4. Results

### 4.1. Descriptive Statistics of the Variables

**Figure 2:**
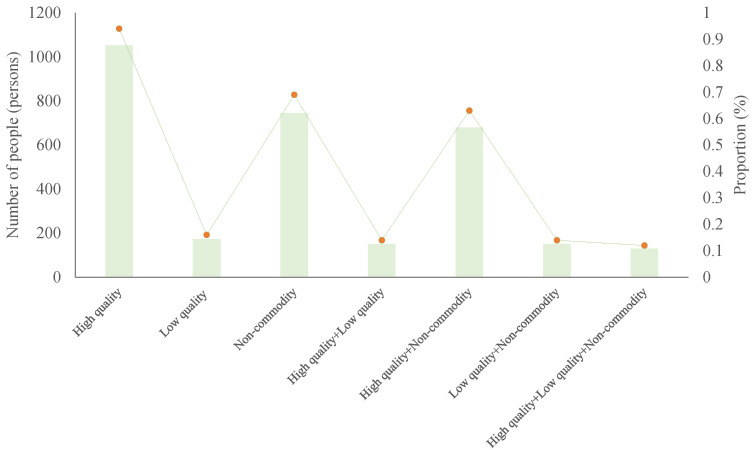
Energy consumption structure.

Figure 2 shows the average number and score of households using seven types of energy in 1080 households. As can be seen from Figure 2, the most households use high-quality energy, accounting for 94%. The least number of households, at 12%, use high-quality, low-quality and non-commercial energy. Among them, the proportion of households using both types of energy is lower than that using one type of energy, and the proportion of households using low quality energy is only 16%, which is far lower than that using high quality and non-commodity energy.

In terms of off-farm employment, overall, rural off-farm employment is 23 per cent. Among the 1080 sample households, 433 households have 0 off-farm employment ratio, and only 15 households have 1 off-farm employment ratio, and the off-farm employment ratio is 0.5 or below, accounting for 91.48% of all sample households.

In terms of control variables, male respondents accounted for 62%, the average age of respondents was about 57 years old, and the average length of education was 6.95 years. Children aged 6 and below and people aged 65 and above accounted for 32% of the total family population. The average annual income of the sample families is 130,000 yuan. The average distance from home to market is 3.9 km, the average farmland area under operation in 2020 is 9.49 mu, and the average building area is 227.7 m^2^. In addition, the respondents’ average cognition score on their own health status was 3.87, and that on climate change was 4.41.

### 4.2. Model Results

Table 2 shows the regression results of off-farm employment of labor force on household energy use. Model 1, Model 3, Model 5, Model 7, Model 9, Model 11 and Model 13 are the benchmark regression results using the Probit model. In order to solve the endogeneity problem, Model 2, Model 4, Model 6, Model 8, Model 10, Model 12 and Model 14 use the regression results of IV-Probit model, and the reported results are the marginal effect of the model and the standard errors are cluster at the county level. 

As shown in Table 2, when the endogenous problem is not dealt with, only the use of high-quality + low-quality energy has a significant correlation with the off-farm employment of labor force. After dealing with the endogenous problem, only the use of high-quality energy has no significant correlation with the off-farm employment of labor force, and the other six types of energy have a significant positive correlation with the off-farm employment of labor force. This result is inconsistent with hypothesis H1. The possible reason is that the use cost of high-quality energy such as electric energy, natural gas and liquefied petroleum gas is relatively high and the use conditions are relatively strict. Therefore, farmers’ use of high-quality energy is mostly influenced by local location conditions, infrastructure, policy guarantee and rural residents’ own family economic conditions. Relatively speaking, off-farm employment of labor force is a weak factor, which is not significantly affected by off-farm employment. In addition, the higher the rural households on the proportion of employment, the more serious the rural hollowing out, to look after the children stay in the rural life or old man, in the traditional ideas, use cost and use under the influence of difficulty, the more willing to use fuel wood, straw and other non-commercial energy or low-quality commodities such as coal, or use power high-quality energy at the same time, but the use of non-commodity and low quality energy still dominates. At the same time, due to the large security risks of natural gas and liquefied petroleum gas, young people who go out to work will also suggest their elders to use biomass energy and low-quality commodity energy, which are easy to obtain and operate, for the safety of their homes. Specifically, the probability of farmers using non-commercial energy will increase by 1.912% on average when the proportion of non-commercial employment of labor force increases by 1%, the highest proportion. When the proportion of off-farm employment of labor force increases by 1%, the probability of farmers using high-quality + low-quality energy will increase by 1.357% on average, with the lowest proportion, but there is no significant difference between the two. In terms of control variables, farmers’ cognition of their own health status, distance from home to market, and area of cultivated land under operation are highly correlated with the use of several types of energy. Specifically, farmers’ cognition of their own health status and cultivated land area are positively and significantly correlated with the use of several types of energy, and the probability of using high-quality energy increases by 11.3% on average with each increase in the health status of farmers. The difference is that the distance between rural households and market is significantly negatively correlated with the use of high-quality energy. The possible reason is that the use of high-quality energy requires not only better economic conditions, but also stricter requirements on local infrastructure such as the opening of natural gas and the purchase of gas tanks, so it also needs better location conditions.

### 4.3. Heterogeneity Analysis

Above, the overall analysis of off-farm employment of labor force on the use of several types of energy by farmers is carried out. It was found that, except for high-quality energy, other types of energy are significantly correlated with off-farm employment of labor force, and the marginal effect coefficients are close. Next, heterogeneity analysis will be carried out on the seven types of energy according to years of education and annual household income, so as to obtain the influence coefficients at each level and understand their correlation in a more detailed way. First of all, the number of years of education is used as a criterion for the following two reasons. On the one hand, with the increase in years of education, farmers’ environmental cognition will change [31,32]. Out of social responsibility and consideration for environmental protection, farmers with different education levels will choose different energy-use schemes. On the other hand, farmers with higher education levels tend to arrange cooking time and cost more efficiently and carefully. The time and cost of using different energy sources are different, so their energy consumption structure will also be affected. In addition, different energy use determines different quality of life. The higher the income level of farmers, the higher their quality of life will be, and they are more willing to use safer, efficient and convenient energy regardless of cost [26]. Therefore, the energy consumption structure of farmers with different incomes often varies to some extent. 

First of all, according to the education years of the respondents, the research objects are divided into two levels: high school or above and high school or below. The IV-Probit method is used for estimation. Table 3 reports the results of grouping regression. The results show that the proportion of off-farm employment in the sample with high school education and above is positively correlated with the use of high-quality energy. Every 1% increase in the proportion of off-farm employment increases the probability of the use of high-quality energy by 4.5% on average, which has the largest promoting effect among several types of energy. This may be because the level of education of farmers will affect their awareness of environmental protection, health and consumption, and promote the proportion of off-farm employment on the use of high-quality energy. This shows that the environmental cognition of rural residents has a great impact on their use of high-quality energy. A total of 953 rural residents have education level below high school, accounting for 88% of the total sample. The use of high-quality energy in this sample is mostly influenced by other factors, and this group plays a decisive role in the selection of high-quality energy in the total sample. Therefore, as mentioned above, the off-farm employment of the total sample is not significantly correlated with the use of high-quality energy. Among the energy categories that include two and three energy sources, there is a positive and significant correlation between the proportion of off-farm employment and the use of low quality + non-commodity energy, except for the sample with high school and above. The possible reason is that for the farmers who still use low quality + non-commodity energy, the most important factors affecting their energy consumption structure are family economic situation, location condition and population structure, rather than the number of years of education, which have no decisive effect on these factors.

Secondly, according to the three equal points of household annual income, the sample is divided into three groups: low income, middle income and high income. IV-Probit method is also used for estimation, and the results are shown in Table 4. The results showed that the proportion of off-farm employment in all three samples was not correlated with the use of high-quality energy, which was consistent with the Results of probit baseline regression and IV-Probit regression. In the high income sample, the proportion of farmers’ off-farm employment is not significantly correlated with the use of non-commodity energy, high quality + non-commodity energy and high quality + low quality + non-commodity energy. The possible reason is that high-income farmers do not have economic pressure, and their use of non-commodity energy is mainly due to family location conditions, traditional habits, population structure and other reasons, while the proportion of off-farm employment does not play a leading role in their use of non-commodity energy. In addition, the proportion of off-farm employment has no significant effect on the use of high-quality energy, and the proportion of off-farm employment in the high-income sample has no significant correlation with the use of high-quality + non-quality energy, and the proportion of off-farm employment in the high-income sample has no significant correlation with the use of high-quality + non-quality energy, and the proportion of off-farm employment in the high-income sample has no significant correlation with the use of high-quality + low-quality + non-commodity energy.

### 4.4. Mechanism Analysis

According to the above theoretical analysis, annual cash income and family population structure were taken as the intermediary variables, respectively, based mainly on the following considerations: On the one hand, labor transfer as an important livelihood strategy, enables migrant workers to increase family income through remittances and other means, thus affecting the overall consumption level of families [20]. With the improvement of consumption level, families gradually prefer convenient and safe living energy [33,34]. On the other hand, studies have shown that the ratio of the elderly to children will affect the decision of the family to go out for employment, and the elderly are more inclined to use traditional biomass energy and other low-quality energy [30], so the dependency/support ratio will also play a role in the off-farm employment of labor force and energy consumption structure.

With annual cash income of the family as the intermediary variable, the following seven paths are mainly verified: Labor employment to families in cash income, high quality/low quality/non-commercial use of energy, labor payrolls to families in cash income, high + low/high + non/low + of energy use, labor payrolls to families in cash income, high low + the energy use, model results are shown in Table 5. Table 5 shows that contains only a single energy in the energy category, cash income families in labor employment and farmers for non-commercial, low quality played a partial mediation effect between energy use, this shows that the improvement of employment proportion will improve family annual cash income reducing for non-commercial farmers and the low quality of energy use. This is in line with the theoretical logic. With the transfer of labor force, many farmers begin to engage in off-farm industries with higher efficiency and higher income, while income is an important factor affecting the energy consumption structure of farmers. With the increase in income, people tend to reduce the use of unsafe and inconvenient energy such as firewood, straw and coal. In the two and three energy categories, annual household cash income plays a partial mediating effect between labor, off-farm employment and household use of these energy categories, and the effect direction is consistent with that of single energy, which verifies research hypothesis H2.

When family population structure is used as an intermediary variable, the following seven paths are mainly verified: off-farm employment of labor → household population structure → high quality/low quality/non-commodity energy use, off-farm employment of labor → household population structure → high + low + non-energy use, off-farm employment of labor → household population structure → high + low + non-energy use, the results of the model are shown in Table 6. Table 6 shows that household demographic structure plays a partial intermediary effect between off-farm employment and farmers’ use of non-commodity, high-quality + non-commodity energy. The corresponding coefficient shows that, with the increase in the proportion of off-farm employment, the ratio of household dependency/support gradually decreases, which further reduces the use of non-commodity and non-commodity + high-quality energy by farmers. With the increase in the dependency/support ratio, farmers’ use of non-commodity and non-commodity + high-quality energy also gradually increased. This may be due to the limited ability to pay and the inherent concept, the elderly are more dependent on traditional solid fuel. For the families with a high proportion of going out to work, the elderly, children and young people are more likely to stay at home, while young people prefer safer and better new energy. This verifies the research hypothesis H3.

## 5. Conclusions and Policy Implications

### 5.1. Conclusions

Based on the questionnaire survey data of 1080 households in Sichuan province in 2021, the study makes an in-depth analysis of the impact of labor off-farm employment on the cooking energy structure of households and its specific mechanism, and draws the following conclusions:

(1) The proportion of off-farm employment of rural households is not high as a whole, only 23%. In the cooking energy structure, the most households use high-quality energy, accounting for 94%; the lowest percentage of households using both high-quality, low-quality and non-commodity energy was 12 percent.
(2) In addition to high-quality energy, off-farm employment of labor force is positively correlated with household energy consumption structures. The probability of farmers using non-commercial energy will increase by 1.912% on average when the proportion of off-farm employment increases by 1%, which is the highest proportion. If the proportion of off-farm employment increases by 1%, the probability of farmers using high-quality + low-quality energy will increase by 1.357% on average, with the lowest proportion. The results of heterogeneity analysis show that the proportion of off-farm employment of farmers with high education level and above has the greatest positive effect on the use of high-quality energy.
(3) The analysis of the mediation effect shows that the off-farm employment can affect the source consumption structure of farmers through two paths: annual cash income and population structure.

### 5.2. Policy Implications

Based on the above conclusions, the research puts forward the following three policy recommendations:

(1) The study found that the increase in the proportion of off-farm employment would promote farmers to use high-quality energy on the basis of non-commodity and low-quality energy. For some developing countries with relatively backward economies, economic development is the focus of social development, and promoting off-farm employment of migrant workers is an important channel to increase income in rural areas and improve the living environment in rural areas. For rural areas in developing countries such as China, it is necessary to strengthen household registration management and social security, and improve the mechanism of equal access to compulsory education and urban housing for children of migrant workers to promote healthy development of the labor market. In addition, reasonable guidance and publicity should be carried out to encourage rural labor to migrate to cities and stabilize the off-farm employment of labor, so as to improve the structure of rural cooking energy use.
(2) Through the mediation effect, it is found that the annual cash income of households significantly promotes the use of high-quality energy by farmers. The economic development of rural areas in Sichuan is relatively backward. Therefore, the local government should improve macro-control, optimize the industrial structure in rural areas, give full play to the advantages of regional industries, encourage farmers to innovate and start businesses and improve farmers’ use of high-quality energy such as natural gas and electricity by increasing their income, so as to promote the transformation and upgrading of the rural energy structure.
(3) Heterogeneity analysis results show that the proportion of off-farm employment has the greatest positive effect on the use of high-quality energy among farmers with higher education levels. At present, the quality of education in rural areas of Sichuan is relatively low, and the gap between urban and rural education is large. Therefore, it is necessary to increase investment in education in rural areas, improve the quality of the rural teaching force and narrow the gap between urban and rural education levels. In addition, it is important to improve the education mechanism in rural areas, guarantee the teaching infrastructure, improve the level of schooling, vigorously supervise and publicize and improve the education level of farmers. According to this, we can strengthen their environmental awareness, environmental awareness, social responsibility and promote their use of high-quality safe, efficient and convenient energy.

## Figures and Tables

**Table 1:** Descriptive statistics.

Variable	Description	Mean	SD
High quality	High quality	0.940	0.240
Low quality	Low quality	0.160	0.360
Non-commodity	Non-commodity	0.690	0.460
High quality + Low quality	High quality + Low quality	0.140	0.350
High quality + Non-commodity	High quality + Non-commodity	0.630	0.480
Low quality + Non-commodity	Low quality + Non-commodity	0.140	0.340
High quality + Low quality + Non-commodity	High quality + Low quality + Non-commodity	0.120	0.320
Labor ratio	Off-farm employment ratio	0.230	0.230
Labor ratio IV	The proportion of off-farm employment of other labor in the same village	0.230	0.120
Annual household cash income	Annual household cash income	130000	380000
Family structure	Dependency/support ratio	0.320	0.280
Gender	Gender (1 = men; 0 = women)	0.620	0.490
Age	Age (years)	57.110	11.220
Edu	Years of schooling (years)	6.950	3.320
Health	Health Status (1–5)	3.870	1.100
Climate	Whether often pay attention to climate change and other information (1–5)	4.410	1.050
Distance	The distance from home to market (m)	3981	6445
Important	How much they attach importance to the honorary titles such as “Civilized sanitation Demonstration Household” and “clean farmer household” by the government (1–5)	3.880	1.210
Land	The area of farmland under operation (Acres)	9.490	61.850
House	The floor area of houses (m^2^)	227.700	144.200

**Table 2:** Effects of Off-farm employment on several types of energy use.

Variables	High Quality Energy		Low Quality Energy		Non-Commercial Energy		High Quality + Low Quality		High Quality + Non-Commodity		Low Quality + Non-Commodity		High Quality + Low Quality + Non-Commodity	
	Model 1	Model 2	Model 3	Model 4	Model 5	Model 6	Model 7	Model 8	Model 9	Model 10	Model 11	Model 12	Model 13	Model 14
Labor ratio	0.510	−0.543	0.399	1.553 ***	0.152	1.912 ***	0.375 **	1.357 ***	0.270	1.588 **	0.186	1.583 ***	0.142	1.414 ***
	(0.476)	(0.742)	(0.257)	(0.289)	(0.161)	(0.730)	(0.192)	(0.228)	(0.195)	(0.633)	(0.236)	(0.350)	(0.159)	(0.346)
Gender	−0.108	−0.116	0.245	0.233	−0.199	−0.158	0.227	0.218	−0.233 **	−0.207 **	0.249	0.234	0.216	0.204
	(0.242)	(0.230)	(0.275)	(0.260)	(0.173)	(0.138)	(0.235)	(0.227)	(0.111)	(0.090)	(0.310)	(0.289)	(0.271)	(0.257)
Age	−0.001	−0.001	−0.002	−0.002	0.004	0.002	0.000	−0.000	0.003	0.002	−0.001	−0.002	0.001	0.000
	(0.008)	(0.007)	(0.005)	(0.005)	(0.006)	(0.005)	(0.004)	(0.004)	(0.005)	(0.004)	(0.005)	(0.005)	(0.005)	(0.004)
Edu	−0.017	−0.018	0.014	0.015	−0.062 **	−0.057 **	0.007	0.008	−0.064 ***	−0.061 ***	0.010	0.011	0.001	0.003
	(0.032)	(0.032)	(0.017)	(0.015)	(0.028)	(0.025)	(0.016)	(0.013)	(0.023)	(0.021)	(0.022)	(0.019)	(0.019)	(0.016)
Health	0.095 **	0.113 ***	0.112 ***	0.081 **	−0.028	−0.057	0.116 **	0.090 **	0.003	−0.022	0.130 ***	0.090 **	0.139 ***	0.104 **
	(0.046)	(0.042)	(0.040)	(0.039)	(0.094)	(0.087)	(0.046)	(0.043)	(0.094)	(0.091)	(0.038)	(0.042)	(0.039)	(0.044)
Climate	0.049	0.059	0.024	0.008	−0.032	−0.049	0.045	0.032	−0.007	−0.022	−0.001	−0.021	0.021	0.003
	(0.059)	(0.057)	(0.033)	(0.029)	(0.093)	(0.078)	(0.041)	(0.038)	(0.088)	(0.079)	(0.048)	(0.045)	(0.059)	(0.058)
Distance	−0.348 *	−0.321 *	0.267 ***	0.221 **	0.335 **	0.265 *	0.198 **	0.160	0.174	0.136	0.342 ***	0.280 **	0.263 *	0.210
	(0.182)	(0.185)	(0.088)	(0.086)	(0.152)	(0.150)	(0.097)	(0.109)	(0.168)	(0.170)	(0.121)	(0.122)	(0.140)	(0.150)
Important	0.061	0.064 *	−0.022	−0.025	−0.098*	−0.091*	−0.013	−0.014	−0.071	−0.069	−0.003	−0.006	0.014	0.011
	(0.039)	(0.033)	(0.060)	(0.055)	(0.053)	(0.049)	(0.067)	(0.063)	(0.054)	(0.051)	(0.054)	(0.049)	(0.057)	(0.053)
Ln(land)	−0.072	−0.092	0.105 **	0.125 ***	0.204 ***	0.224 ***	0.076	0.095 *	0.185 ***	0.205 ***	0.099 **	0.124 ***	0.089 *	0.113 **
	(0.086)	(0.072)	(0.049)	(0.044)	(0.040)	(0.051)	(0.052)	(0.050)	(0.028)	(0.038)	(0.042)	(0.041)	(0.054)	(0.052)
Ln(house)	0.117 *	0.147 **	0.159 *	0.097	0.034	−0.038	0.195 **	0.140	0.059	0.005	0.131	0.056	0.149 *	0.079
	(0.067)	(0.062)	(0.093)	(0.105)	(0.131)	(0.149)	(0.094)	(0.105)	(0.110)	(0.124)	(0.113)	(0.116)	(0.085)	(0.088)
Control/County	Yes	Yes	Yes	Yes	Yes	Yes	Yes	Yes	Yes	Yes	Yes	Yes	Yes	Yes
Chi2		27.614		108.008		1187.448		73.390		19.919		764.799		222.130

Note: Robust standard errors in parentheses; * *p* < 0.1, ** *p* < 0.05, *** *p* < 0.01.

**Table 3:** Heterogeneity analysis results: Years of education.

Variables	High Quality Energy/High Quality + Low Quality/High Quality + Low Quality + Non-Commodity		Low Quality Energy/High Quality + Non-Commodity		Non-Commercial Energy/Low Quality + Non-Commodity	
	Under the High School	High School and Above	Under the High School	High School and Above	Under the High School	High School and Above
A. A kind of energy						
Labor ratio	−1.093	4.500 ***	1.692 ***	1.164	1.811 ***	2.760
	(0.779)	(0.312)	(0.240)	(1.094)	(0.591)	(2.051)
Chi2	32.779	843.460	118.144	81.469	159.812	61.495
B. Two kinds of energy						
Labor ratio	1.371 ***	2.325 ***	1.309 ***	3.948 ***	1.735 ***	0.693
	(0.235)	(0.820)	(0.455)	(1.118)	(0.291)	(1.217)
Chi2	110.586	252.283	35.537	277.312	327.761	16.327
C. Three kinds of energy						
Labor ratio	1.445 ***	2.030 **				
	(0.321)	(0.847)				
Chi2	151.545	271.439				
County	Yes	Yes	Yes	Yes	Yes	Yes
Control	Yes	Yes	Yes	Yes	Yes	Yes
N	953	127	953	127	953	127

Note: Robust standard errors in parentheses; ** *p* < 0.05, *** *p* < 0.01.

**Table 4:** Heterogeneity analysis results: Annual household cash income.

Variables	High Quality Energy/High Quality + Low Quality/High Quality + Low Quality + Non-Commodity			Low Quality Energy/High Quality + Non-Commodity			Non-Commercial Energy/Low Quality + Non-Commodity			
	Low Income	Middle Income	High Income	Low Income	Middle Income	High Income	Low Income	Middle Income	High Income	
A. A kind of energy										
Labor ratio	−1.364	−0.481	0.541	1.848 ***	1.802 ***	1.527 ***	2.656 ***	1.638 **	2.244	
	(0.945)	(1.277)	(0.847)	(0.488)	(0.553)	(0.339)	(0.747)	(0.808)	(1.441)	
chi2	74.981	5.141	9.784	149.352	359.164	38.157	57.303	100.046	10.675	
B. Two kinds of energy										
Labor ratio	1.451 ***	2.043 ***	0.877 **	1.668 ***	1.329 *	2.082	1.837 ***	1.857 **	1.663 **	
	(0.436)	(0.504)	(0.411)	(0.565)	(0.779)	(1.382)	(0.400)	(0.858)	(0.657)	
chi2	21.068	185.855	15.685	171.080	17.075	48.518	359.494	391.676	48.040	
C. Three kinds of energy										
Labor ratio	1.439 ***	2.111 **	1.103							
	(0.380)	(0.822)	(0.758)							
Chi2	26.121	218.783	16.294							
Control	Yes	Yes	Yes	Yes	Yes	Yes	Yes	Yes	Yes	Yes
County	Yes	Yes	Yes	Yes	Yes	Yes	Yes	Yes	Yes	Yes
N	360	360	360	360	360	360	360	360	360	360

Note: Robust standard errors in parentheses; * *p* < 0.1, ** *p* < 0.05, *** *p* < 0.01.

**Table 5:** Mechanism of the impact of Off-farm employment on several types of energy use: annual household cash income.

Variables	Off-Farm → Income → Non-Commercial/Low Quality + Non-Commodity/ High Quality + Low Quality + Non-Commodity			Off-Farm → Income → Low Quality/High Quality + Non-Commodity			Off-Farm → Income → High Quality Energy/ High Quality + Low Quality		
	Non-Commercial/Low Quality + Non-Commodity/ High Quality + Low Quality + Non-Commodity	Ln (Income)	Non-Commercial /Low Quality + Non-Commodity/ High Quality + Low Quality + Non-Commodity	Low Quality/High Quality + Non-Commodity	Ln (Income)	Low Quality/High Quality + Non-Commodity	High Quality/ High Quality + Low Quality	Ln (Income)	High Quality/ High Quality + Low Quality
A. A kind of energy									
Off-farm employment	1.912 *** (0.730)	2.001 *** (0.360)	2.348 *** (0.909)	1.553 *** (0.289)	2.001 *** (0.360)	1.783 *** (0.280)	−0.543 (0.742)	2.001 *** (0.360)	−0.826 (0.866)
Ln (Income)			−0.240 *** (0.086)			−0.139 *** (0.024)			0.163 *** (0.062)
B. Two kinds of energy									
Off-farm employment	1.583 *** (0.350)	2.001 *** (0.360)	1.839 *** (0.401)	1.588 ** (0.633)	2.001 *** (0.360)	1.880 ** (0.831)	1.357 *** (0.228)	2.001 *** (0.360)	1.542 *** (0.253)
Ln (Income)			−0.156 *** (0.034)			−0.166 * (0.085)			−0.113 *** (0.026)
C. Three kinds of energy									
Off-farm employment	1.414 *** (0.346)	2.001 *** (0.360)	1.618 *** (0.433)						
Ln (Income)			−0.123 *** (0.045)						
County/Control	Yes			Yes			Yes		

Note: Robust standard errors in parentheses; * *p* < 0.1, ** *p* < 0.05, *** *p* < 0.01.

**Table 6:** Mechanism of the impact of Off-farm employment on several types of energy use: family demographic structure.

Variables	Off-Farm → Family Structure → Non-Commercial/Low Quality + Non-Commodity/High Quality + Low Quality + Non-Commodity			Off-Farm → Family Structure → Low Quality/ High Quality + Non-Commodity			Off-Farm → Family Structure → High Quality/ High Quality + Low Quality		
	Non-Commercial/Low Quality + Non-Commodity/ High Quality + Low Quality + Non-Commodity	Family Structure	Non-Commercial/Low Quality + Non-Commodity/ High Quality + Low Quality + Non-Commodity	Low Quality/High Quality + Non-Commodity	Family Structure	Low Quality/High Quality + Non-Commodity	High Quality/ High Quality + Low Quality	Family Structure	High Quality/ High Quality + Low Quality
A. A kind of energy									
Off-farm employment	1.912 *** (0.730)	−0.311 ** (0.092)	1.814 *** (0.558)	1.553 *** (0.289)	−0.311 ** (0.092)	1.502 *** (0.280)	−0.543 (0.742)	−0.311 ** (0.092)	−0.477 (0.632)
Family structure			0.714 ** (0.278)			−0.113 (0.255)			−0.449 (0.392)
B. Two kinds of energy									
Off-farm employment	1.583 *** (0.350)	−0.311 ** (0.092)	1.528 *** (0.318)	1.588 ** (0.633)	−0.311 ** (0.092)	1.512 *** (0.506)	1.357 *** (0.228)	−0.311 ** (0.092)	1.311 *** (0.225)
Family structure			0.008 (0.307)			0.491 *** (0.154)			−0.203 (0.187)
C. Three kinds of energy									
Off-farm employment	1.414 *** (0.346)	−0.311 ** (0.092)	1.362 *** (0.319)						
Family structure			−0.092 (0.235)						
County/Control	Yes			Yes			Yes		

Note: Robust standard errors in parentheses; ** *p* < 0.05, *** *p* < 0.01.

## Data Availability

Not applicable.
